# A genetically identified population of layer 4 neurons in auditory cortex that contributes to pre-pulse inhibition of the acoustic startle response

**DOI:** 10.3389/fncir.2022.972157

**Published:** 2022-09-08

**Authors:** Aldis P. Weible, Iryna Yavorska, Arthy Narayanan, Michael Wehr

**Affiliations:** Department of Psychology, Institute of Neuroscience, University of Oregon, Eugene, OR, United States

**Keywords:** gap detection, auditory cortex, layer 4 circuits, optogenetic activation, cortical microcircuitry

## Abstract

A fundamental task faced by the auditory system is the detection of events that are signaled by fluctuations in sound. Spiking in auditory cortical neurons is critical for sound detection, but the causal roles of specific cell types and circuits are still mostly unknown. Here we tested the role of a genetically identified population of layer 4 auditory cortical neurons in sound detection. We measured sound detection using a common variant of pre-pulse inhibition of the acoustic startle response, in which a silent gap in background noise acts as a cue that attenuates startle. We used a Gpr26-Cre driver line, which we found expressed predominantly in layer 4 of auditory cortex. Photostimulation of these cells, which were responsive to gaps in noise, was sufficient to attenuate the startle reflex. Photosuppression of these cells reduced neural responses to gaps throughout cortex, and impaired behavioral gap detection. These data demonstrate that cortical Gpr26 neurons are both necessary and sufficient for top–down modulation of the acoustic startle reflex, and are thus likely to be involved in sound detection.

## Introduction

Understanding how cortical circuitry contributes to sensory perception and behavioral output is one of the central goals of systems neuroscience. Pursuit of this goal has benefited tremendously from advances in the ability to manipulate and record from identified cell types in behaving animals (for review, see Adesnik and Naka, 2018). Here we focus on gap detection, a behavioral temporal processing paradigm that involves primary auditory cortex, and that provides a robust and quantitative behavioral output. Gap detection serves as a model for temporal processing challenges faced by the auditory system, such as the detection and identification of speech sounds, species-specific vocalizations, or other events signaled by acoustic fluctuations in the environment (Plomp, 1964; Green, 1985). Here we measured gap detection using a common variant of pre-pulse inhibition (PPI) of the acoustic startle response, in which silent gaps inserted into continuous background noise act as cues to attenuate the startle reflex. PPI is a fundamental form of sensorimotor gating in the brain, which is impaired in a number of neuropsychiatric disorders (Koch, 1999). Both ascending and descending auditory pathways in the brainstem, midbrain, and cortex contribute to PPI (for review, see Li et al., 2009; Lauer et al., 2017). Although auditory cortex is not required for conventional PPI, it is essential for the detection of brief gaps (≤32 ms in duration) (Ison et al., 1991; Kelly et al., 1996; Bowen et al., 2003; Threlkeld et al., 2008; Masini et al., 2012; Weible et al., 2014b,2020b). We recently found that a layer 3 → 5 circuit in auditory cortex contributes to gap detection (Weible et al., 2020a), but how these and other elements of the cortical microcircuit interact to mediate this behavior remains unknown.

The canonical cortical microcircuit, first proposed for the visual system (Gilbert and Wiesel, 1983; Douglas and Martin, 1991), was an early attempt at describing the flow of information through cortex. In this model, sensory information from the thalamus first enters cortex in layer 4 (L4), then projects to superficial layers L2/3, then to deep layers L5/6, from where it either exits cortex or closes the loop via a L6 → L4 projection. Theoretically, to understand how information is transformed as it passes through the circuit, one could measure neuronal responses at each node, and test how manipulations at one node impact responses at the next. Because perceptual gap detection is mediated by gap-evoked spiking responses in cortical neurons (Weible et al., 2014b), if the underlying canonical cortical microcircuit is serial, it would predict that L4 neurons should be necessary and sufficient for gap detection.

Extensive study and the application of increasingly sophisticated tools have revealed far greater complexity within cortical microcircuits than originally envisaged. Thalamocortical inputs have been found terminating in all layers of cortex (for review, see Harris and Shepherd, 2015; Williamson and Polley, 2019). Whereas the canonical circuit described a largely serial progression of information, there is now evidence of numerous interconnections between layers and sublayers (Douglas and Martin, 2004; for reviews, see Adesnik and Naka, 2018). Furthermore, cells once classified as excitatory or inhibitory have been further differentiated by morphology, physiology, and patterns of connectivity, suggesting functional specialization. Recognition of these advances has prompted the suggestion that cell types, rather than layers, may serve as the relevant nodes within the circuit (Harris and Shepherd, 2015; Gerfen et al., 2018; Williamson and Polley, 2019). This suggests that in addition to understanding the role of cortical layers, recording from and manipulating specific cell classes, such as genetically identified cell types, may be a useful strategy for understanding how cortical circuitry contributes to sensorimotor computation.

Here we examined the role of a genetically identified class of cells in gap detection. We used the Gpr26-Cre driver line KO250 (Gerfen et al., 2013), which has not previously been characterized in auditory cortex. We found that Gpr26 cells were predominantly located in layer 4. A subset of these cells responded to gaps, with gap duration tuning that is typical for auditory cortical neurons. Photosuppression of Gpr26 cells reduced gap-evoked responses across layers in auditory cortex, and impaired behavioral gap detection. Photoactivation of Gpr26 cells was sufficient to attenuate behavioral startles, much like acoustic gaps in noise. Thus Gpr26 neurons are both necessary and sufficient for top-down cortical modulation of the acoustic startle reflex.

## Materials and methods

### Mice

All procedures were performed in accordance with National Institutes of Health guidelines, as approved by the University of Oregon Institutional Animal Care and Use Committee. We used + / + offspring (8–12 weeks of age) of crosses between hemizygous Tg(Gpr26-Cre)KO250Gsat (“Gpr26”; 036915-UCD; MMRRC) and homozygous CAG-ChR2-eYFP (“ChR2”; 012569, Ai32, The Jackson Laboratory), or CAG-Arch-eGFP (“Arch”; 012735, Ai35D, The Jackson Laboratory), or Rosa-CAG-LSL-tdTomato-WPRE (“tdTomato”; Ai14, 007914, The Jackson Laboratory) lines. In these offspring, Cre-dependent Channelrhodopsin-2 (ChR2; behavior: *n* = 7 mice; physiology: *n* = 4 mice), Archaerhodopsin (Arch; behavior: *n* = 5; physiology: *n* = 5), or tdTomato (*n* = 6) was expressed in Cre-expressing, Gpr26 pyramidal neurons. The Gpr26 gene encodes G protein-coupled receptor 26 (Lee et al., 2000). For behavioral and electrophysiological experiments, we used Gpr26-Cre-negative littermates as controls (behavior: *n* = 11; physiology: *n* = 8). We used Gpr26-tdTomato (*n* = 3) and Gpr26-Cre-negative (*n* = 8) littermates for fluorescent retrobead injections.

### Anatomy

Mice were perfused transcardially with 0.1 M phosphate buffered saline (PBS) followed by 4% paraformaldehyde in PBS (60 ml, 2 ml/min). Brains were removed and post-fixed for an additional 24 h in 4% paraformaldehyde, then sectioned at 50 μm in the coronal plane. We used six Gpr26-tdTomato mice (8–12 weeks of age), and took photomicrographs of alternating sections on a Zeiss microscope using Zen software (Carl Zeiss Microscopy GmbH 2011). Seven of these photomicrographed sections from each mouse were matched to the closest representative atlas section (Paxinos and Franklin, 2004). We selected a rectangular region oriented perpendicular to the cortical surface, with a height extending from the pia to the external capsule, and a width 1/8th of this height, through the middle of primary auditory cortex (A1). To establish laminar boundaries, we subdivided this rectangular region approximating the findings of Anderson et al. (2009): layers 1, 2, 3, and 4 each represented 12.5% of the cortical thickness, and layers 5 and 6 each represented another 25%. We further subdivided each layer into two equal sublayers, to obtain finer-grained measures of penetrance with depth. A sample count of cells was taken from the rectangular region. Counts of tdTomato-labeled cells were taken from 7 coronal sections for each mouse, at ∼200 μm spacing. For three of the mice, we then performed *in situ* hybridization on the sections to label putative pyramidal neurons positive for Ca+/calmodulin protein kinase II (CaMKII). We used a digoxygenin (DIG)-labeled riboprobe (1:500), visualized by Anti Fluor-POD (1:1000; Invitrogen, Cat. A21253) and Fluorescein (1:50; PerkinElmer, Cat. NEL741), as described previously (Weible et al., 2014b). We were not able to test for co-localization of tdTomato and CaMKII at cellular resolution, because *in situ* hybridization quenched the fluorescent tdTomato signal and also slightly distorted the tissue, which prevented precise spatial alignment of before-and-after images. We therefore quantified CaMKII-labeled cells across lamina, using the same rectangular regions, in order to measure the penetrance of Gpr26 cells as a proportion of all excitatory cells. We also compared Gpr26-tdTomato fluorescence to the distribution of cortical neurons projecting to dorsal inferior colliculus (dIC) and the medial geniculate body of the thalamus (MGB). To do this, we injected retrobeads into dIC or MGB as described below (see section “Retrobead injection”), waited 2 weeks, and then quantified retrobead signal in auditory cortex by sublamina as described above. We performed retrobead injections in 3 Gpr26-tdTomato mice (green retrobeads only) and 8 Gpr26-Cre-negative mice (red and green retrobeads). In Gpr26-tdTomato mice, we first identified labeled cells separately in the green (retrobead) and red (tdTomato) channels, and then merged the images to assess co-localization.

### Retrobead injection

We administered atropine (0.03 mg/kg) pre-surgically to reduce respiratory irregularities. Mice were anesthetized with isoflurane (1.25–2.0%). We used fluorescent retrobeads (Lumafluor) and performed injections (45 nl, 15 nl/min) through craniotomies in the skull into either the dIC (AP: –5.0 mm, ML: 0.8 mm, DV: 0.8 mm) or the MGB (AP: –3.3 mm, ML: 1.8 mm, DV: 3.0 mm). The injection needle (Hamilton Microliter #7000.5) was maintained at depth for 5 min following each injection, and then slowly withdrawn. The craniotomy was filled with anti-bacterial ointment and the skull covered with dental cement. We administered ketoprofen (4.0 mg/kg) post-operatively. Mice were housed communally following surgery.

### Fiber implantation

We administered atropine (0.03 mg/kg) pre-surgically to reduce respiratory irregularities. Mice were anesthetized with isoflurane (1.25–2.0%). One craniotomy was drilled in each hemisphere dorsal to auditory cortex (AP: –2.9 mm, ML: 4.4 mm, relative to bregma) for the placement of 200 μm-diameter optic fibers (on the pial surface). We used cyanoacrylate and dental cement to secure the fibers to the skull. We administered ketoprofen (4.0 mg/kg) post-operatively. Mice were housed communally following surgery and were given 7 days to recover.

### Behavioral data acquisition and stimulus delivery

Methods are as described previously (Weible et al., 2014a,2020a,2020b). All behavioral data were collected in a sound-attenuating chamber. Sounds were delivered from a free-field speaker directly facing the animal. The speaker was calibrated to within ± 1 dB using a Brüel and Kjaer 4939 microphone positioned where the ear would be during behavioral sessions. Mice were loosely restrained in a plastic tube (35 mm inner diameter, 1.5 mm wall thickness) affixed to a flat base. The tube was perforated (∼3 mm diameter holes) to allow effective transmission of sound, with no more than 5 dB attenuation. The head was loosely clamped in position. An open slot along the top enabled access to the implanted fibers. Startle responses were measured with a piezo transducer positioned beneath the tube.

We inserted silent gaps into continuous 80 dB SPL background white noise, and measured how these gaps attenuated startle responses elicited by a 100 dB SPL, 25 ms white noise burst. Gaps were 2, 4, 8, 16, or 32 ms in duration, and were separated from the startle stimulus by a 50 ms post-gap interval. We also presented startle stimuli in isolation, not preceded by a gap (“gap-free” trials) to provide a baseline startle response. Each combination of gap duration and light condition (see below) was presented 20 times per session, randomly interleaved and separated by a randomized inter-trial interval of 15 ± 5 s.

We separately examined how photostimulation and photosuppression of Gpr26 cells affected behavior. For photostimulation we used mice expressing Channelrhodopsin-2 (Gpr26-ChR2) and 445 nm wavelength laser diodes set to an output power of 50, 100, 200, or 300 mW/mm^2^. For photosuppression we used mice expressing Archaerhodopsin (Gpr26-Arch) and 520 nm wavelength laser diodes with an output power of 300 mW/mm^2^. We chose these intensities based on previous characterization of their spatial spread in auditory cortex (Weible et al., 2014a,b). Light-On trials were pseudorandomly interleaved with Light-Off trials. For photosuppression of gap-evoked cortical activity in Gpr26-Arch mice, we delivered a 50 ms light pulse that started at gap termination and ended at startle stimulus onset (see inset in Figure 3A). For photoactivation in Gpr26-ChR2 mice, we delivered a 25 ms pulse that started 50 ms prior to startle stimulus onset on gap-free trials only (see inset in Figure 5). To visually mask photostimulation, we used strobe lights equipped with blue or green filters that pulsed continuously for the duration of the session.

### Behavioral analysis

We quantified startle response amplitudes by calculating the area of the rectified startle signal within a 100 ms window following onset of the startle stimulus. To evaluate PPI, only sessions with significant gap detection for at least one gap duration (Light-Off) were included for analysis, based on a significance test comparing startle amplitudes associated with each gap duration to startle amplitudes on gap-free trials (paired *t*-test, *p* < 0.05). Behavioral curves were based on the median startle attenuation at each gap duration. We used non-parametric tests for group analyses because data for some gap durations were not normally distributed (Lilliefors test), and because statistical power is comparable even when the underlying assumptions for the corresponding parametric analysis is met (Kitchen, 2009). We used the Kruskal–Wallis test (K–W; non-parametric alternative to the one-way Anova) for comparisons between Light-On and Light-Off conditions across gap durations. For analysis of Gpr26-ChR2 data, we used the Wilcoxon rank-sum test to compare median Light-On startle responses between genotypes (Light-On responses normalized to the session’s median Light-Off response). Gap detection data were collected and analyzed from the same mouse for no more than five sessions, to minimize the likelihood of introducing experience-related shifts in startle behavior at brief gap durations (Swetter et al., 2010).

### Electrophysiology

Mice were anesthetized with isoflurane (1.25–2.0%). A headpost was secured to the skull and a mark was made on the skull over auditory cortex for a future craniotomy (AP: –2.9 mm, ML: 4.4 mm, relative to bregma). Mice were housed individually following surgery and were allowed at least 5 days of post-operative recovery. On the day of recording, mice were anesthetized with isoflurane (1.25–2.0%), the head was fixed in position using the headpost, and a small craniotomy was made over auditory cortex (1 × 1 mm). Craniotomies were covered with a thin layer of agar and mice were allowed to recover for at least 1 h before recording.

All electrophysiological recordings were performed while the animal was awake and head-fixed on a rotating, spindle-mounted styrofoam ball inside a double-walled acoustic isolation booth. We recorded neurons in auditory cortex with a 32-channel silicon probe (25 μm spacing between sites, 750 μm shank, Neuronexus A1 × 32-Poly2-5mm-50s-177), using an Intan RHD2132 headstage and RHD2000 acquisition board, and Open Ephys software (Siegle et al., 2017). The silicon probe was positioned with a micromanipulator (MP-285, Sutter) orthogonal to the cortical surface such that the electrode sites spanned cortical layers. Spiking and local field potential data were filtered online (600–6000 Hz and 0.1–400 Hz, respectively). We identified single neurons offline using MClust spike sorting software (Redish, 2008) analogously to tetrode recordings (Weible et al., 2014b,2020b). To do this, we grouped contiguous recording sites into groups of four, and then used peak and trough waveform voltage, energy, and principal components analysis as waveform separation parameters in 2-dimensional cluster space. Cells were accepted for analysis only if they had a cluster boundary completely separate from adjacent cluster boundaries, and completely above threshold, on at least one 2-D view. Additionally, cells with events during a 2 ms refractory window in the interspike interval histogram in excess of 0.5% of the total spike count were excluded from analysis. To measure the depth of recorded cells, we used current-source density analysis of the local field potential evoked by 25 ms white noise bursts delivered once per second. We identified the robust sink with the shortest latency at the L3–L4 boundary and assigned it a depth of 400 μm (Intskirveli and Metherate, 2012; Weible et al., 2020a). We assigned the depths of individual neurons relative to this, based on the channel exhibiting the maximum waveform amplitude for each neuron. This allowed us to relate recording depth to our histological analysis and laminar boundaries (Anderson et al., 2009). For analyses of multi-unit activity, high-pass filtered spiking activity was assigned to a single cluster for each tetrode, to which we assigned the depth corresponding to the most superficial channel of each tetrode.

We collected neural responses to gap-in-noise stimuli (Gpr26-Arch and Gpr26-ChR2), as well as to 445 nm light pulses for photoidentification of Gpr26 neurons (Gpr26-ChR2 only). The presentations of gap-in-noise stimuli differed from the behavioral protocols in two respects. First, no startle stimuli were presented. Second, a shorter inter-trial interval was used (1 s instead of the 15 ± 5 s used during behavioral experiments). Recording sessions included 20 presentations each of gaps 1, 2, 4, 8, 16, and 32 ms in duration, as well as 20 gap-free trials. Gap termination responses (GTRs) were defined as a significant increase (paired *t*-test) in spiking activity during the 50 ms post-gap interval (i.e., following the resumption of noise) for at least two consecutive gap durations relative to the same interval during gap-free trials (Weible et al., 2014b,2020a,2020b). All other comparisons were performed using non-parametric analyses because some involved non-normally distributed data (Lilliefors test). The effect of illumination on spiking activity for each cell was expressed as a *z*-value (from the Wilcoxon rank-sum test statistic). We used the Kruskal–Wallis (K–W) test to assess within-group effects of illumination (in *z*-values) across depths. We used the Wilcoxon rank-sum test for *post hoc* comparisons to the K–W test, as well as for group comparisons (transgenic versus control). We used a Bonferroni correction for multiple comparisons where appropriate. We report effect sizes as eta-squared (η^2^) (Lenhard and Lenhard, 2016). η^2^ varies between 0 and 1, and corresponds to the proportion of variance in the dependent variable explained by the independent variable. η^2^ values of 0.01 – 0.06 are generally considered to be small effects, η^2^ of 0.06 – 0.14 moderate effects, and η^2^ > 0.14 large effects.

We used light pulses to attempt to identify putative Gpr26 neurons in Gpr26-ChR2 mice, as described previously (Lima et al., 2009; Weible et al., 2020a). A train of blue light pulses (445 nm, 5 ms duration) was delivered at a frequency of 10 Hz for 1 s. Twenty repetitions of this train were presented. We characterized light-evoked responses using four measures: response significance, response signal-to-noise, peak response latency, and response reliability (adapted from Lima et al., 2009). We measured response significance as the *p*-value of a paired *t*-test comparing spiking activity during the 25 ms following onset of each light pulse to an equivalent light-free baseline interval. We measured response signal-to-noise as the *z*-value generated from a rank-sum test of the same data. We measured response latency as the time-to-peak of the Gaussian-smoothed (5 ms S.D.) trial-averaged firing rate following the onset of each light pulse. We measured response reliability as the proportion of trials on which light evoked 1 or more spikes in a 50 ms window following the onset of each light pulse. We classified cells as “Photoresponsive” if they met either of two sets of criteria: (1) significance *p* < 0.0001, peak latency < 20 ms, and reliability > 0.5, or (2) significance *p* < 0.0001 and peak latency < 15 ms.

### Histology

Following behavioral and electrophysiological experiments, we coronally sectioned (50 μm) all brains and confirmed the presence or absence of transgene expression (based on eGFP or eYFP fluorescence) in auditory cortex.

## Results

The detection of brief gaps in noise involves auditory cortex, and specifically relies on the GTR (a burst of spikes evoked during the post-gap interval) (Ison et al., 1991; Kelly et al., 1996; Bowen et al., 2003; Threlkeld et al., 2008; Weible et al., 2014b,2020b), but the circuit mechanisms by which this activity contributes to gap detection remain unknown. Here we tested the role of a layer-specific, genetically identified class of pyramidal cells in this behavior. First, we characterized the expression pattern of the Gpr26-Cre line in A1, which we found expressed mainly in layer 4. We then examined how suppressing or stimulating Gpr26 cells influenced GTRs and behavioral gap detection.

### Gpr26 cells are found predominantly in layer 4

**FIGURE 1:**
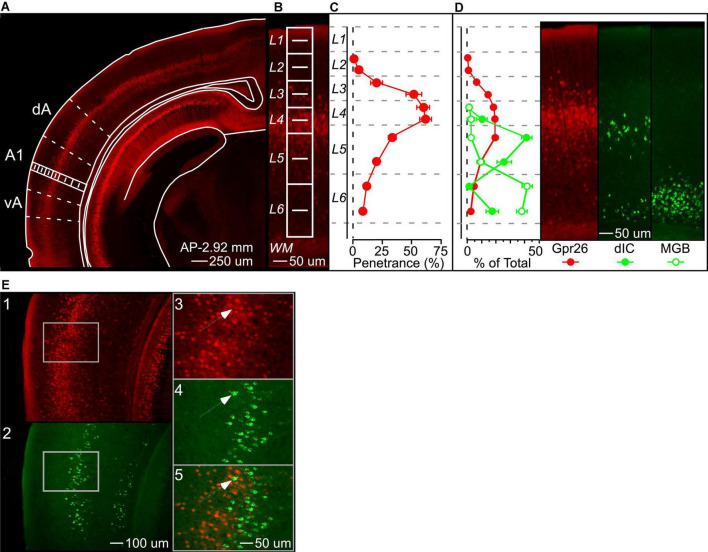
Gpr26 cells form a dense band in layer 4 of auditory cortex. **(A)** We visualized Gpr26 expression by crossing the Gpr26-Cre driver line to a Cre-dependent Td-tomato fluorescent reporter line. 25X magnification. **(B)** To quantify penetrance, we counted Td-tomato labeled cells in a rectangular region (in white) extending from the pial surface through layer 6 of primary auditory cortex (A1), with layers subdivided into two equal halves for increased granularity. Laminar divisions were adapted from Anderson et al. (2009). *In situ* hybridization enabled visualization and quantification of Ca2 + /calmodulin-dependent protein kinase II positive (CaMKII +) excitatory neurons (not shown). Penetrance was defined as the ratio of Gpr26 to CaMKII + cells. 100X magnification. **(C)** Penetrance was greatest in layer 4 (62%), and included 52% of excitatory cells across middle layers 3b-5a. **(D)** Fluorescent retrobeads injected into dorsal inferior colliculus (dIC) and the medial geniculate body of the thalamus (MGB) labeled cortical output neurons in layers 5&6. Percentages reflect the proportion of labeled cells in each laminar subdivision relative to the total number of labeled cells across subdivisions, for each group. 100X magnification. **(E)** Co-localization of Gpr26-tdTomato and dIC-injected retrobead fluorescence was limited to approximately 1% of cells across layers 4b-5b, the layers of greatest overlap of the two populations. To determine the extent to which Gpr26 neurons contributed to the dIC projection, fluorescent Gpr26 (panel 1) and dIC-projecting (panel 2) neurons were counted across layers 4b-5b of A1. Images were then merged to quantify co-localization of identified neurons from the two channels. As illustrated, cells fluorescing in both the Td-tomato red (panel 3) and retrobead green (panel 4) channels would appear yellow (panel 5) when merged (see arrow). 50X and 100X magnification. dA and vA, dorsal and ventral auditory cortex fields; WM, white matter. Coronal outlines adapted from Paxinos and Franklin (2004). Plots show mean ± S.E.

Expression in the Gpr26-Cre line in auditory cortex has not previously been described in detail. The original characterization of this line reported specific layer 5 expression in most cortical areas (Gerfen et al., 2013). To confirm this for auditory cortex, we quantified the laminar expression pattern and penetrance of Gpr26 cells in A1. We counted labeled cells across layers in Gpr26-tdTomato mice (Figures 1A,B, *n* = 6 mice), and then counted CaMKII-positive (excitatory) cells in the same sections labeled by *in situ* hybridization. We did not measure whether individual cells showed co-localization of tdTomato and CaMKII (due to tissue processing effects; see section “Materials and Methods”), and instead quantified penetrance as the percentage of labeled Gpr26 cells relative to the total number of excitatory neurons in each layer. In contrast to the original report, we found that the density of Gpr26 cells in A1 was highest in layer 4, and dropped off gradually across layers 3 and 5 (Figure 1C). Penetrance in layer 4 reached 62%. Superficial to layer 4, penetrance in layer 3b was 52%, declining to 22% in layer 3a. In layer 5, 35% of layer 5a cells expressed tdTomato, declining to 22% in layer 5b. Only layer 1 appeared to be completely devoid of tdTomato fluorescence.

### Gpr26 cells do not project to thalamus or inferior colliculus

Penetrance of the Gpr26 line is thus predominantly in layer 4 of auditory cortex, raising the possibility that these cells could be recipients of thalamic input and could be involved in the initial stages of cortical processing. However, we also observed Gpr26 cells in layer 5, traditionally considered an output layer in the canonical cortical circuit. We therefore wondered whether Gpr26 cells in layer 5 make output projections. Two major recipients of deep-layer auditory cortical output are the dIC and the MGB of the thalamus. We injected green fluorescent retrobeads into dIC and MGB (in separate animals) to compare the laminar distribution of corticofugal cells with tdTomato-labeled Gpr26 cells. Retrobeads injected into the dIC of eight mice (3 Gpr26-Ai14 and 5 Gpr26-Cre-negative) labeled two bands of cells, one predominantly in layer 5a and thus partially overlapping with the population of Gpr26 cells, and the other in layer 6b (Figure 1D). Injections in MGB terminated in the ventral subdivision (MGBv). However, fluorescence was observed along the track, passing through the dorsal subdivision, suggesting that retrograde transport from MGBd cells may have occurred in addition to MGBv (data not shown). Retrobeads injected into the MGB of seven mice resulted in dense labeling spanning layers 6a and 6b, and thus shared very little spatial overlap with Gpr26 cells. These patterns of retrograde transport from dIC and MGB are consistent with previous reports (Winer et al., 1998, 2001; Winer, 2006; Malmierca and Ryugo, 2011).

Because the more superficial band of dIC-projecting neurons overlapped spatially with tdTomato-labeled Gpr26 cells, we looked for co-localization in three Gpr26-tdTomato mice with dIC green retrobead injections. As indicated in Figure 1D, the superficial band of dIC projecting cells spanned layers 4b-5b. To maximize the likelihood of identifying co-labeled cells, we quantified all fluorescent Gpr26 cells and retrobead labeled cells throughout A1 layers 4b-5b (seven sections from each of three mice). Labeled cells were identified by eye separately in the green (retrobead) and red (tdTomato) channels first, and then the images were merged to assess co-localization. Of the 3030 fluorescent Gpr26 cells and 1025 cells labeled with green fluorescent retrobeads identified across 21 sections from three mice, co-localization was observed in just 30 cells (for example, Figure 1E, panel 5). Thus, approximately 1% of Gpr26 cells appeared to project to dIC. These results indicate that, despite penetrance extending into deep layers of cortex, the Gpr26 line appears to provide minimal contribution to either of these two main cortical output pathways.

### Suppression of Gpr26 cells impairs gap detection

#### Validation of photosuppression

Because Gpr26 cells were primarily found in layer 4, the main input layer to auditory cortex, we hypothesized that they might contribute to gap responses throughout the cortical column, as well as to behavioral gap detection. To test this with photosuppression, we generated a Gpr26-Arch cross that expressed Archaerhodopsin in Gpr26 neurons, and illuminated auditory cortex during the post-gap interval, when GTRs of A1 neurons occur. First, we validated our photosuppression method by recording from 336 A1 neurons in 5 Gpr26-Arch mice using silicon probes. To accurately determine the depth of recorded neurons, we used current-source density analysis to identify a sound-evoked robust short-latency sink at the L3/L4 border (Intskirveli and Metherate, 2012). Our analyses focused on the 280 cells from sessions for which we could unambiguously determine laminar depth. For comparison, we analyzed activity of 210 cells with verified depths recorded from 4 Gpr26-Cre-negative littermate control mice.

#### Suppression of baseline firing

**FIGURE 2:**
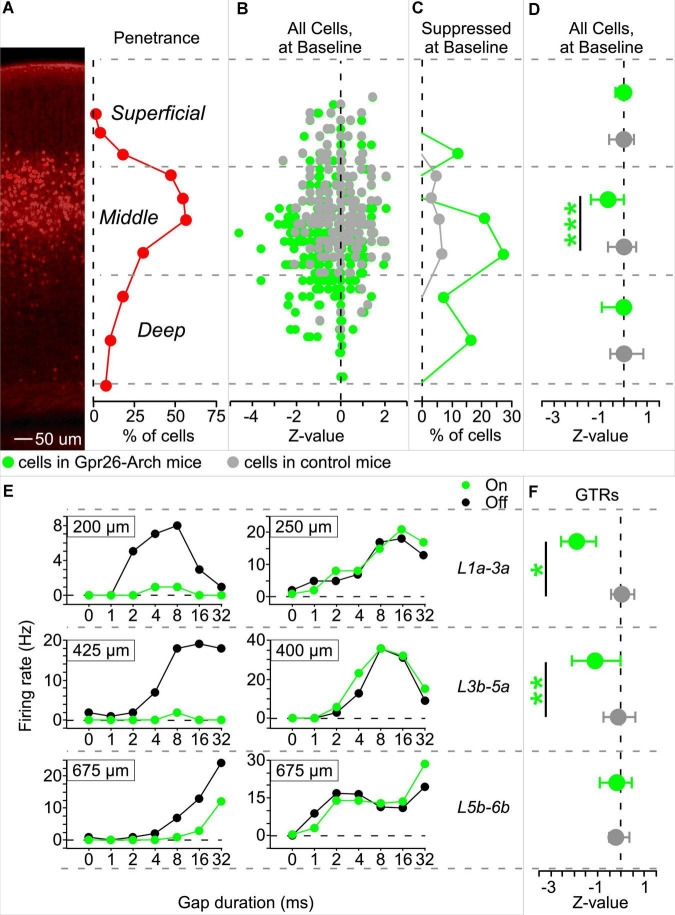
Illumination suppresses gap termination responses (GTRs) in Gpr26-Arch mice. Spiking activity was recorded using silicon probes during the delivery of gap-in-noise stimuli. Analyses of spiking activity were limited to those cells for which depth could be unambiguously determined, based on current source density analysis (Gpr26-Arch: 280 cells; control: 210 cells). Illuminated (Light-On) trials were interleaved with non-illuminated (Light-Off) trials. Illumination occurred during the 50 ms interval following gaps, and the corresponding interval during gap-free baseline trails. **(A)** Cells were separated by depth into three groups, those within the range of greatest penetrance of the Gpr26 line (middle layers 3b-5a), and those dorsal and ventral to that range (superficial layers 1a-3a and deep layers 5b-6b, respectively). 100X magnification. **(B)** *Z*-values were calculated (Light-On versus Light-Off) for baseline, gap-free trials and then plotted by depth for cells from Gpr26-Arch mice (in green) and controls (in gray). **(C)** The highest proportion of cells from Gpr26-Arch mice suppressed during gap-free trials were found in the middle layers. Proportions represented the number of cells suppressed in each sublayer divided by the total cells recorded in the sublayer. **(D)** Suppression was significant in middle layer cells from Gpr26-Arch mice relative to controls. **(E)** Gap duration tuning curves from 6 example cells. Each panel shows firing rate across different gap durations. Green: photosuppression, black: control. In Gpr26-Arch mice, some GTRs from superficial, middle, and deep layers were suppressed (left-hand column) while others were unaffected (right-hand column). **(F)** At the population level, GTRs from middle and superficial layers, but not deep layers, were significantly suppressed relative to controls. Dots show medians and error bars show IQR. *p < 0.05; **p < 0.01; ***p < 0.0001, rank-sum.

To validate the effectiveness of our photosuppression method, we first tested whether it reduced baseline firing rates of cells at the expected cortical depth (i.e., predominantly layer 4). To see how suppression was related to depth, we sorted cells into middle layers 3b-5a (the sublayers of greatest penetrance), superficial layers 1a-3a, and deep layers 5b-6b (Figure 2A). Figure 2B illustrates the effect of illumination on baseline activity as a function of depth for each cell recorded in Gpr26-Arch and control mice. The shift to the left in cells from Gpr26-Arch mice (in green) relative to cells from control mice (in gray) shows that illumination suppressed baseline spiking activity, especially in the middle layers. The proportion of significantly photosuppressed cells in the middle layers was 24% (41/173, Figure 2C), compared with 7% (1/14) and 9% (8/93) in the superficial and deep layers, respectively. Few cells from controls showed any effect of illumination (Figure 2C). For cells from Gpr26-Arch mice, there was a trend toward depth as a main effect for suppression (Table 1). Overall, suppression was highly significant relative to controls (Table 1), with the greatest effect observed in the middle layers (Figure 2D and Table 1).

**FIGURE 3:**
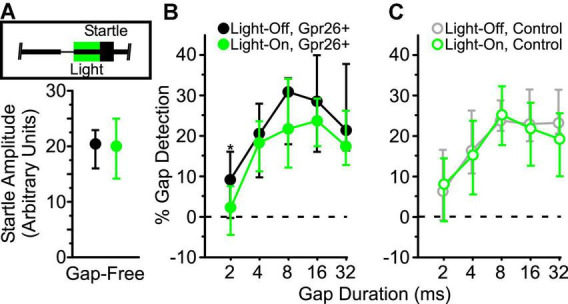
Suppression of Gpr26 neurons reduces behavioral gap detection. Light (520 nm, 300 mW/mm^2^) was delivered to auditory cortex through chronically-implanted optic fibers during the 50 ms interval preceding the startle (inset in **A**). Behavioral data were collected from Gpr26 (*n* = 5, 19 sessions) and control (*n* = 4, 12 sessions) mice. **(A)** No difference in pure startle amplitude (gap-free trials) was observed with illumination for Gpr26 mice (*p* = 0.87, η^2^ = 0.001). To compare gap detection across durations, we normalized startles to the median non-illuminated gap-free startle. **(B,C)** Illumination significantly reduced gap detection in Gpr26 mice, but had no impact on controls. Boxes show median and IQR. * *p* < 0.05, rank-sum *post hoc*.

**TABLE 1:** Effects of illumination on cells in transgenic versus control mice.

All cells	Test	*P*-values	η^2^	χ^2^
Gpr26 Suppression × Depth	K–W	0.07	0.01	5.2
Gpr26 × Controls, All Depths	Rank-sum	<0.0001	0.04	
Superficial	Rank-sum	0.86	0.001	
Middle	Rank-sum	<0.0001	0.06	
Deep	Rank-sum	0.08	0.03	
**GTR Cells**				
Gpr26 Suppression × Depth	K–W	0.005	0.08	10.5
Gpr26 × Controls, All Depths	Rank-sum	0.004	0.05	
Superficial	Rank-sum	0.02	0.36	
Middle	Rank-sum	0.006	0.07	
Deep	Rank-sum	0.71	0.004	

Values in black are statistically significant, values in gray are not.

#### Suppression of gap termination responses

In Gpr26-mice, illumination during the post-gap interval suppressed GTRs in some cells, but not others, across superficial, middle, and deep layers (see examples in Figure 2E). Of the 280 cells for which we established a reliable measure of depth, 40% exhibited GTRs (113/280). The proportion of cells exhibiting GTRs at each depth was similar (superficial layers: 7/14 or 50%; middle layers: 71/173 or 41%; deep layers: 35/93 or 38%). Illumination significantly suppressed 28% of recorded GTRs (32/113). How effectively illumination suppressed these GTRs varied significantly by depth (Figure 2F and Table 1), with significant population-level photosuppression in the middle and superficial layers but not in the deep layers (Figure 2F and Table 1).

#### Impairment of gap detection

To test whether suppression of these cells directly impacted behavior, we illuminated auditory cortex during the 50 ms post-gap interval, or during a corresponding control interval on gap-free trials, preceding the delivery of an acoustic startle stimulus (Figure 3A, inset). Illumination had no effect on startle responses for gap-free trials (Figure 3A), ruling out a non-specific effect on startle responses. On trials with a gap, illumination significantly reduced gap detection in Gpr26-Arch mice (Figure 3B; *p* = 0.03, η^2^ = 0.03, rank-sum; 5 mice, 19 sessions). No effect of illumination was observed on behavior in Gpr26-negative littermate control mice (Figure 3C; 4 mice, 12 sessions), ruling out any non-specific effects of light delivery on behavior. These results indicate that Gpr26 cells are involved in gap detection behavior.

### Stimulation of Gpr26 cells directly attenuates the startle response

Our finding that Gpr26 cells contribute to behavioral gap detection prompts two key predictions. First, at least some of these cells should respond to gaps. Second, their direct activation should attenuate startles even in the absence of a gap (mimicking gap detection). We tested these predictions in Gpr26-ChR2 mice that expressed Channelrhodopsin-2 in Gpr26-Cre + neurons.

#### Validation of photoactivation

**FIGURE 4:**
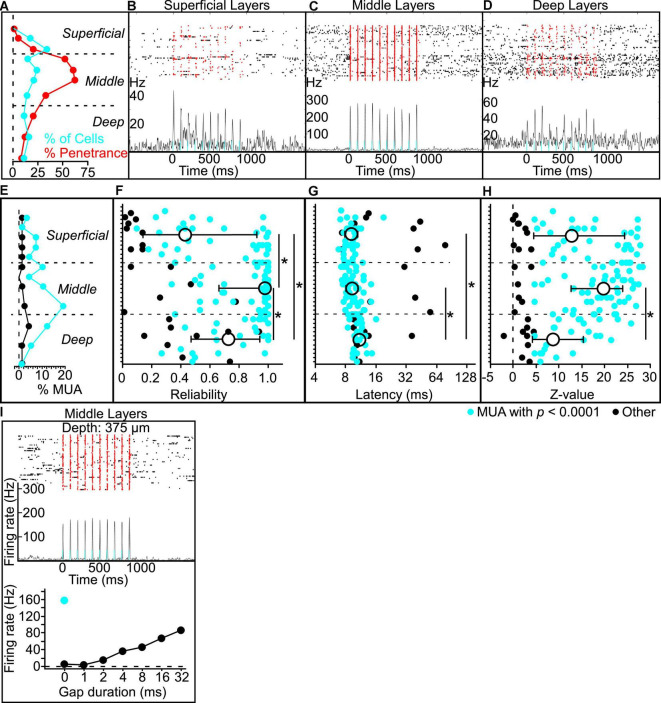
Putative Gpr26 neurons exhibit gap termination responses (GTRs). **(A)** The percentage of total cells recorded per sublayer that were found to be photoresponsive (in blue) was far lower than expected based on the penetrance of the line as determined in GPR26-tdTomato mice (in red, same as Figure 1C). However, when applied to multi-unit activity (MUA), the same analyses yielded results consistent with the Gpr26 expression pattern. **(B–D)** Example multi-unit recordings from a single silicon probe penetration (approximate depths **B:** 175 μm, **C:** 475 μm, **D:** 875 μm). The raster (top) displays spikes before, during, and after each pulse train presentation. Red dots indicate spikes captured during presentations of each 5 ms light pulse of each train. Trial-averaged firing rate is shown below the raster plots; note the different vertical scales for firing rate. **(E)** Using a conservative significance threshold for MUA (*p* < 0.0001; see section “Materials and methods”), a higher proportion of all photoresponsive “tetrodes” (see section “Materials and methods”) recorded was found in middle layers 3b-5a compared with superficial and deep layers (layers 1a-3a and layers 5b-6b, respectively). MUA analyses in E-H included significantly responsive (blue) and non-responsive (black) tetrodes**. (F)** Median reliability in middle layers exceeded that observed in superficial and deep layers. Reliability in the superficial layers was also lower than that in the deep layers. **(G)** Median peak response latency in both middle and superficial layers was faster than that observed in deep layers. **(H)** Signal-to-noise (*z*-values) in middle layers was greater than that observed in deep layers, with a trend toward more robust responses versus superficial layers. **(I)** Activity of an example middle-layer putative Gpr26 neuron, including a robust phase-locked response to the pulse train (top panel), GTRs evoked by multiple gap durations (bottom panel, black dots), and a robust increase in spiking with illumination during gap-free trials (bottom panel, blue dot). Boxes show median and IQR. *p < 0.05, rank-sum.

To attempt to photoidentify Gpr26 neurons, we recorded from 277 cells from 4 Gpr26-ChR2 mice. Our analyses focused on the 241 cells for which we could unambiguously determine laminar depth based on current source density analysis. For comparison, we analyzed activity from 156 cells with verified depths recorded from 4 Gpr26-Cre-negative littermates that did not express ChR2. We observed fewer photoresponsive cells than expected, with a markedly different laminar distribution than that expected from the anatomical expression pattern (Figure 4A). We suspect that this could be due to strong activation of a dense excitatory population, which may have interfered with spike-sorting of our extracellular recordings. We used a specific set of criteria to identify photoresponsive cells (see section “Materials and Methods: Electrophysiology”). Of 241 cells, only 36 (or 15%) qualified as directly photoresponsive. In the middle layers (Figure 4A), only 17% of cells were photoresponsive (19/114), which is threefold less than we would expect from the 52% penetrance in middle layers that we observed in Gpr26-tdTomato mice. Although we and others have successfully used this photoidentification method before (Lima et al., 2009; Moore and Wehr, 2013, 2014; Weible et al., 2014a,2020a), those studies used lines with either inhibitory populations or with a lower expression density of excitatory cells than we report here for Gpr26. We wondered whether simultaneous photoactivation of the relatively high proportion of Gpr26 cells within the middle layers could have distorted extracellular spike waveforms, resulting in spikes during the light pulse being missed by spike-sorting. To test this idea, we instead analyzed recordings as multi-unit activity, by pooling extracellular threshold-crossing events without spike-sorting. We found that light evoked robust and reliable multi-unit activity in the middle layers, but comparatively weaker activation in the superficial and deep layers (see examples in Figures 4B–D). More recording sites in the middle layers had significant light-evoked multi-unit activity (Figure 4E) compared with superficial and deep layers. Median reliability, peak latency, and signal-to-noise of light-evoked responses all differed significantly across superficial, middle, and deep layers (Figures 4F–H and Table 2). Reliability was greatest in the middle layers, followed by the deep layers. Peak response latency in both middle and superficial layers was briefer than that observed in deep layers. Signal-to-noise (*z*-values) was greatest in the middle layers, followed by the superficial layers. Multi-unit activity recorded in control mice did not show light-evoked activity (data not shown). These results are consistent with the anatomical penetrance of the mouse line, indicating that ChR2 is indeed expressed by Gpr26 neurons in the expected depth range, and that light robustly photoactivates these neurons. These results are also consistent with the interpretation that spikes from a considerable fraction of Gpr26 single neurons were missed by spike-sorting during photoactivation.

**FIGURE 5:**
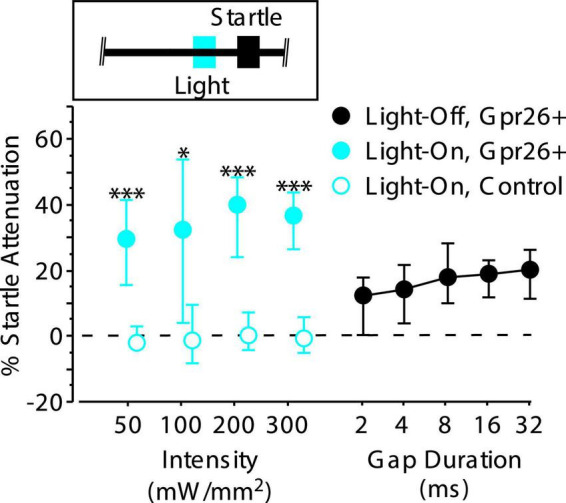
Stimulating Gpr26 cells directly attenuates the startle reflex. Light pulses, 25 ms in duration, were delivered 50 ms prior to startle onset (see inset) during gap-free trials. Four different intensities were tested. Each intensity elicited significant attenuation of the startle reflex, with median attenuation at the lowest intensity exceeding that elicited by a 32 ms gap (for intensities 50, 100, 200, and 300 mW/mm^2^: *n* = 6 mice, 13, 16, 20, and 14 sessions, respectively). No attenuation was observed with illumination in control mice (for intensities 50, 100, 200, and 300 mW/mm^2^: *n* = 5 mice, 12, 15, 21, and 13 sessions, respectively). Boxes show median and IQR. *p < 0.01; ^***^p < 0.0001, rank-sum.

**TABLE 2:** Variability of reliability, peak latency, and signal-to-noise by depth.

	Test	*P*-value	η^2^	χ^2^
Reliability	K–W	<0.0001	0.16	22.5
Middle × Superficial	Rank-sum	<0.0001	0.18	
Middle × Deep	Rank-sum	0.003	0.09	
Superficial × Deep	Rank-sum	0.04	0.06	
Peak Latency	K–W	0.0002	0.11	17.1
Middle × Superficial	Rank-sum	0.45	0.006	
Middle × Deep	Rank-sum	0.0001	0.15	
Superficial × Deep	Rank-sum	0.0006	0.16	
Signal-to-Noise	K–W	0.0005	0.10	15.2
Middle × Superficial	Rank-sum	0.12	0.02	
Middle × Deep	Rank-sum	<0.0001	0.18	
Superficial × Deep	Rank-sum	0.13	0.03	

Multiple comparisons threshold: p < 0.017.

Values in black are statistically significant, values in gray are not.

#### Gpr26 cells respond to gaps

Although we were only able to photoidentify a subset of Gpr26 cells, we still wondered whether any of those cells responded to gaps. Of the 36 putative Gpr26 neurons described above, more than half (21/36 or 58%) responded to gaps with a GTR. The response of one such cell to both photostimulation and gaps in noise is illustrated in Figure 4I. This demonstration that Gpr26 cells are gap responsive is consistent with the gap detection deficit produced by photosuppression (Figure 3A), leading us to test our second prediction that photoactivation of these cells should also mimic the behavioral effect of gaps, attenuating startle responses.

#### Effects of photostimulation on behavior

To test whether photoactivation could influence the PPI pathway, we delivered 25 ms light pulses beginning 50 ms before the startle stimulus on gap-free trials (see Figure 5, inset). Photoactivation strongly attenuated the startle reflex of Gpr26-ChR2 mice compared to controls across a wide range of light intensities (Figure 5; 50 mW/mm^2^*: p* < 0.0001, η^2^ = 0.72; 100 mW/mm^2^: *p* = 0.002, η^2^ = 0.32; 200 mW/mm^2^: *p* < 0.0001, η^2^ = 0.54; 300 mW/mm^2^: *p* < 0.0001, η^2^ = 0.59, rank-sum). Startle attenuation at the lowest light intensity was stronger than that produced by the longest (most effective) gap duration tested. Illumination had no effect in control mice not expressing ChR2, ruling out artifactual attenuation due to visual or intracranial detection of the light pulses. Thus, photoactivation of Gpr26 cells alone was sufficient to modulate startle responses.

## Discussion

Here we examined the involvement of a layer-specific, genetically identified class of pyramidal neurons in auditory cortex in PPI of the acoustic startle response. We found that Gpr26 neurons were predominantly located in layer 4, and are likely recipients of thalamic input. Photosuppression of Gpr26 cells suppressed spiking activity across layers in A1 and impaired behavioral gap detection. Gpr26 cells were responsive to gap-in-noise stimuli, and photoactivation of these cells was sufficient to attenuate the startle reflex. This startle attenuation was robust even at the lowest light intensity tested, and closely resembled that evoked by acoustic gaps in noise. To our knowledge, this is the first characterization of this Gpr26 line in auditory cortex, and provides insights into the role of these layer 4 cells in sound processing.

In rodents, Gpr26 mRNA expression has been detected in numerous structures throughout the brain (Lee et al., 2000). Several Gpr26-Cre lines have been generated with distinct expression profiles. The line we used (KO 250) has previously been characterized as expressing in cortical layer 5 (among other regions) and as contributing to both the intratelencephalic tract (IT) that projects intracortically as well as the pyramidal tract (PT) pathway that projects subcortically (Gerfen et al., 2013; The Gensat Project, n.d.). By crossing this Gpr26 line with a Cre-dependent tdTomato fluorescent reporter line, we found in auditory cortex that penetrance was most dense in layer 4, with just over half of middle layer 3b-5a cells expressing Gpr26. We found minimal co-localization of tdTomato with cells retrogradely labeled from either the MGB of the thalamus or dIC, two major recipients of corticofugal projections from auditory cortex (Winer et al., 1998, 2001; Winer, 2006). Thus Gpr26 cells do not appear to project to MGB or dIC, suggesting that they are mainly IT rather than PT neurons. These observations suggest that Gpr26 cells are involved early in the cortical microcircuit, either as direct recipients of thalamic input or during the initial synaptic processing of sound. We cannot, however, rule out the possibility that a small subset of these cells might project subcortically. Our thalamic retrobead injections primarily targeted the ventral MGB. Injections targeting dorsal MGB might be more likely to reveal any corticofugal-projecting Gpr26 cells in layer 5b, if they exist (Ramaswamy and Markram, 2015; for reviews, see Adesnik and Naka, 2018). Cortico-collicular projections are heaviest to dIC, but weaker projections have also been described to the external cortex and central nucleus of the IC (for review, see Schofield, 2010). Furthermore, Gpr26 cells in layer 5 could project via the IT pathway to other cortical regions (Koralek et al., 1990), or via the PT pathway to structures such as the auditory striatum (LeDoux et al., 1991; Hunnicutt et al., 2016; Ponvert and Jaramillo, 2019).

Our analyses of neuronal activity, performed using silicon probes that enabled verification of recording depth, were consistent with the anatomical penetrance we observed in Gpr26-tdTomato mice. Illumination in the Gpr26-Arch cross significantly suppressed spiking activity in the middle layers, corresponding to the depth of greatest penetrance, and superficial layers 1a-3a, immediately downstream in the canonical circuit. In the Gpr26-ChR2 cross, illumination elicited significant increases in more multi-unit activity sites in middle layers compared with those in superficial or deep layers 5b-6b. The Gpr26-ChR2 recordings also revealed higher reliability and signal-to-noise in middle layers than at adjacent depths, and both middle layers and superficial layers exhibited briefer peak response latencies versus those observed further downstream in the canonical circuit in deep layers.

We found that photoidentification of individual Gpr26 neurons was only partially successful, providing an important cautionary note about this method. We expected, based on penetrance of the Gpr26 line (Figure 1) as well as previous experience (Weible et al., 2020a), that half of middle-layer cells would be photoresponsive. Surprisingly, fewer than a quarter met our criteria. Given the clear evidence of photostimulation in our multi-unit recordings, this suggests that light-evoked spikes for many single-neuron recordings were missed by spike-sorting. We suspect that waveform distortion of spikes occurring during photoactivation could have interfered with spike-sorting based on extracellular waveforms. At least three mechanisms could contribute to this interference: (1) extracellular spike overlap of many simultaneously spiking neurons, (2) altered extracellular waveform shape due to local resistivity changes in the extracellular space from a barrage of synaptic conductance, and/or (3) altered intracellular waveform shape due to paroxysmal depolarization. In epilepsy patients, these mechanisms are known to interfere with spike sorting from neurons in the ictal core during seizure events (Merricks et al., 2015). Because 62% of cells express Gpr26 in layer 4, a layer with strong recurrent connectivity (Binzegger et al., 2004), it seems likely that photoactivation of these cells would produce considerable spike overlap along with widespread synaptic conductance and strong depolarization among neighboring cells. Paroxysmal depolarization also produces waveforms of decreasing amplitude and distorted shape riding atop a large depolarization (Traub and Wong, 1982). All of these types of distortions would be expected to challenge any spike-sorting algorithm that is based on extracellular waveforms, suggesting that photoidentification of any dense population of recurrently connected excitatory neurons (such as Gpr26 cells) would face difficulties. In contrast, it’s unlikely that the photoelectric effect interfered with our photoidentification method. Although light (especially coherent light) can under some conditions generate voltage transients on recording electrodes that resemble action potentials (Kozai and Vazquez, 2015), these would be expected to be locked to light onset, to artifactually increase rather than decrease the number of photoresponsive cells, and to be most prominent at the cortical surface and decrease with depth, none of which we observed in our data.

Our results are somewhat at odds with another recent study utilizing optogenetics to examine interlaminar connectivity. In that study (Pluta et al., 2015), optogenetic suppression of layer 4 modestly reduced activity in more superficial layers. This is consistent with our findings that GTRs were suppressed in superficial layers (Figure 2F). However, Pluta also observed disinhibition in layer 5, attributed to a disynaptic translaminar inhibitory L4–L5 circuit. We did not observe this in our data. However, their approach used a different Cre line (scnn1-tg3-Cre) with expression far more tightly restricted to layer 4 (in somatosensory cortex). Thus the absence of layer 5 disinhibition in our results could be due to the slightly broader expression pattern in the Gpr26-Cre line, or could be due to scnn1-tg3-Cre and Gpr26-Cre targeting distinct populations of layer 4 cells, or both.

Our results demonstrate that optogenetic stimulation and suppression of a genetically identified subset of cortical neurons in layer 4 can modulate the acoustic startle reflex. Layer 4 neurons primarily target superficial layers 2 and 3 (in addition to making recurrent excitatory connections within layer 4) (Harris and Shepherd, 2015). We recently found that layer 3 neurons send a strong projection to layer 5, which in turn inhibits startle responses through the powerful corticocollicular projection from layer 5 to IC (Bajo and King, 2012; Weible et al., 2020a,b). The picture that emerges from these findings is that layer 4 may influence startle responses through a chain of translaminar synaptic processing (layer 4 → 3 → 5) followed by a corticofugal projection from layer 5 → IC. Behavioral effects produced solely by optogenetic stimulation have been described previously (Carrillo-Reid et al., 2019; Marshel et al., 2019; Chong et al., 2020; Weible et al., 2020a; Luis-Islas et al., 2021), and raise the intriguing question of how such stimulation is perceived by the animal. In the context of PPI, the ability of a pre-pulse stimulus (of any modality) to inhibit the startle response is tightly correlated with the extent to which that pre-pulse is consciously perceived (Fendt et al., 2001). For this reason, PPI has often been interpreted as an index of perception. For example, the demonstration of PPI elicited by electrical stimulation of the dorsal cochlear nucleus was interpreted as evidence that such stimulation induces hearing (Zhang and Zhang, 2010). However, electrical stimulation of multimodal structures such as superior colliculus or the pedunculopontine tegmental nucleus can also elicit PPI, and it seems less clear that stimulation of these structures would produce an acoustic percept *per se*. Nevertheless, behavior may be driven by electrical or optogenetic stimulation of remarkably small populations of sensory cortical neurons (Houweling and Brecht, 2008; Huber et al., 2008), and intracortical electrical stimulation of auditory cortex in humans can evoke the auditory perception of sounds (Penfield and Perot, 1963; Fenoy et al., 2006). It therefore seems conceivable that optogenetic activation of neurons in auditory cortex could evoke a phantom acoustic percept. It may be possible to test this in future studies using an operant paradigm, such as a 2-alternative forced choice task, with illumination of auditory cortex delivered in such a way as to generate discriminable responses (Huber et al., 2008; Yang et al., 2008).

## Data availability statement

The raw data supporting the conclusions of this article will be made available by the authors, without undue reservation.

## Ethics statement

The animal study was reviewed and approved by the University of Oregon Institutional Animal Care and Use Committee.

## Author contributions

AW designed and performed the experiments, analyzed the data, and wrote the manuscript. IY performed the electrophysiology experiments and analyzed the data. AN performed the behavioral experiments. MW conceived, designed, and directed research, and helped write the manuscript. All authors contributed to the article and approved the submitted version.
